# Evaluation of Factors Associated with Dual Use of E-Cigarettes in University Students

**DOI:** 10.1093/eurpub/ckac129.036

**Published:** 2022-10-25

**Authors:** FN Öznur Muz, S Metintaş, MF Önsüz, S Aydoğan Gedik, A Mutlu, T Arslan Torba

**Affiliations:** 1 Public Health, Eskisehir Osmangazi University Faculty of Medicine, Eskisehir, Turkey; 2 Public Health, Odunpazari District Health Directorate, Eskisehir, Turkey

## Abstract

**Background:**

The World Health Organization defined smoking as the fastest spreading and longest lasting epidemic globally. It has been reported that two-thirds of all tobacco consumption in the world is in developing countries, and with today's technology, the use of electronic cigarettes (e-cigarettes) has increased rapidly among young people and adults, especially in recent years. The study aimed to evaluate the factors associated with dual use of e-cigarettes (e-cigarette plus one of the tobacco products) in university students.

**Methods:**

This study was carried out with the participation of 2477 students at Eskişehir Osmangazi University in the 2019-2020 academic year, and it was designed as a nested case-control study from a study in which 49 were determined to be dual smokers. A randomized 1:3 for age and gender with 147 non-smokers (NS) and 147 classic cigarette smokers (CSS) selected by the propensity score matching method was performed, with the final sample consisting of 343 participants. Chi-square and multinomial logistic regression analyzes were used in the study.

**Results:**

In the multinomial logistic regression, the belief that e-cigarettes do not help quit classical cigarette smoking was 4.0 (95% CI; 1.7 - 9.6) times higher in NS and 4.1 (1.6 - 10.0) times higher in CCS compared to dual smokers, while the belief that e-cigarettes may suppress the desire to smoke was 4.4 (1.7 - 11.2) times higher in NS and 6.8 (2.6 - 17.6) times higher in CCS.

**Conclusions:**

The study determined that dual smokers were less likely to believe that e-cigarettes are more innocent than other tobacco products. While dual smokers believed that e-cigarettes might not suppress the desire to smoke, CCS believed e-cigarettes could even increase classical cigarette smoking.

**Key messages:**

• Since the effects of e-cigarette use on human health are controversial, it is still a significant public health problem in developing countries.

• Although it was initially marketed to help quit or reduce the use of classical cigarettes, it should be noted that e-cigarettes are also a type of tobacco product addiction.

